# Micronutrient interactions: Magnesium and its synergies in maternal–fetal health

**DOI:** 10.1002/fsn3.4316

**Published:** 2024-07-17

**Authors:** Vani Shukla, Sidrah Parvez, Ghizal Fatima, Shikha Singh, Aminat Magomedova, Gaber El‐Saber Batiha, Athanasios Alexiou, Marios Papadakis, Nermeen N. Welson, Najah Hadi

**Affiliations:** ^1^ Department of Food and Nutrition, Faculty of Nutritional Sciences Era University Lucknow India; ^2^ Department of Biotechnology Era University Lucknow India; ^3^ Department of Population Lomonosov Moscow State University Moscow Russia; ^4^ Department of Pharmacology and Therapeutics, Faculty of Veterinary Medicine Damanhour University Damanhour AlBeheira Egypt; ^5^ Department of Science and Engineering Novel Global Community Educational Foundation Hebersham New South Wales Australia; ^6^ AFNP Med Wien Austria; ^7^ University Centre for Research & Development Chandigarh University Mohali Punjab India; ^8^ Department of Research & Development Funogen Athens Greece; ^9^ Department of Surgery II, University Hospital Witten‐Herdecke University of Witten‐Herdecke Wuppertal Germany; ^10^ Department of Forensic Medicine and Clinical Toxicology, Faculty of Medicine Beni‐Suef University Beni Suef Egypt; ^11^ Department of Pharmacology and Therapeutics, Faculty of Medicine University of Kufa Kufa Iraq

**Keywords:** fetal, magnesium, maternal

## Abstract

Magnesium is an essential nutrient for various physiological processes and becomes even more vital during pregnancy, contributing to muscle relaxation, bone development, electrolyte balance, and blood pressure regulation. Despite the fact that the dietary sources of magnesium are diversified, it is still challenging to obtain it in sufficient quantities during pregnancy. We have elucidated its interactions and its specific impact on maternal–fetal health in different research publications. Magnesium also interacts synergistically with other micronutrients like calcium, vitamin D, potassium, zinc, iron, and vitamin B6, emphasizing its significance in promoting optimal outcomes for both the mother and the fetus. Inadequate magnesium intake during pregnancy has been linked to complications like gestational diabetes, preeclampsia, preterm birth, and low birth weight. Therefore, there should be an emphasis on the importance of maintaining adequate magnesium levels through a balanced diet and supplementation. This research therefore studies the complex mechanisms of micronutrient interactions and their specific impacts on maternal–fetal health to enhance prenatal care practices.

## Graphical abstract

Magnesium is an essential nutrient for various physiological processes and becomes even more vital during pregnancy, contributing to muscle relaxation, bone development, electrolyte balance, and blood pressure regulation. Magnesium interacts synergistically with other micronutrients like calcium, vitamin D, potassium, zinc, iron, and vitamin B6, emphasizing its significance in promoting optimal outcomes for both the mother and the fetus. Inadequate magnesium intake during pregnancy has been linked to complications like gestational diabetes, preeclampsia, preterm birth, and low birth weight.

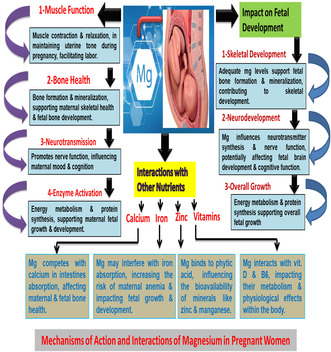

## 1 INTRODUCTION

Maternal–fetal health is an important concern for the development of a nation. Maternal complications can trigger a cascade of adverse outcomes, including preterm birth, low birth weight, developmental issues, and maternal health challenges. Optimal maternal nutrition exerts profound impacts on both fetal growth and birth outcomes. It is considered a modifiable risk factor that can be particularly beneficial in reducing adverse birth outcomes, especially in low‐income populations. Maternal dietary choices and nutritional status substantially influence the course of pregnancy, notably with regard to fetal birth weight (Dunn, 2019).

A healthy diet prior to conception lowers the risk of congenital anomalies. A healthy, well‐nourished woman has a higher probability of giving birth to a baby who will have a better chance of developing into a healthy child and healthy adult. Maternal nutrition during pregnancy affects placental development and fetal growth as well. Micronutrient demands rise during the course of pregnancy, and deficiencies may result from their loss or malabsorption, insufficient intake, lack of knowledge about prenatal nourishment, or the practice of food or dietary taboos during pregnancy, which can be detrimental to both the mother and the fetus.

One micronutrient that has garnered increasing attention in the context of maternal–fetal health is magnesium. Magnesium is a mineral that is integral to a wide array of vital physiological functions in the human body. During pregnancy, its significance becomes even more pronounced as it actively contributes to the development and maintenance of maternal and fetal health and helps in muscle relaxation, bone development, electrolyte balance, blood pressure regulation, etc. Despite the critical role of magnesium, pregnant women often face challenges in meeting their magnesium requirements. The increased nutrient demands associated with pregnancy, coupled with factors like morning sickness and dietary preferences, can hinder magnesium intake. Thus, the need to explore magnesium's role in pregnancy and its interactions with other essential micronutrients becomes apparent (Davenport et al., 2018).

**FIGURE 1:**
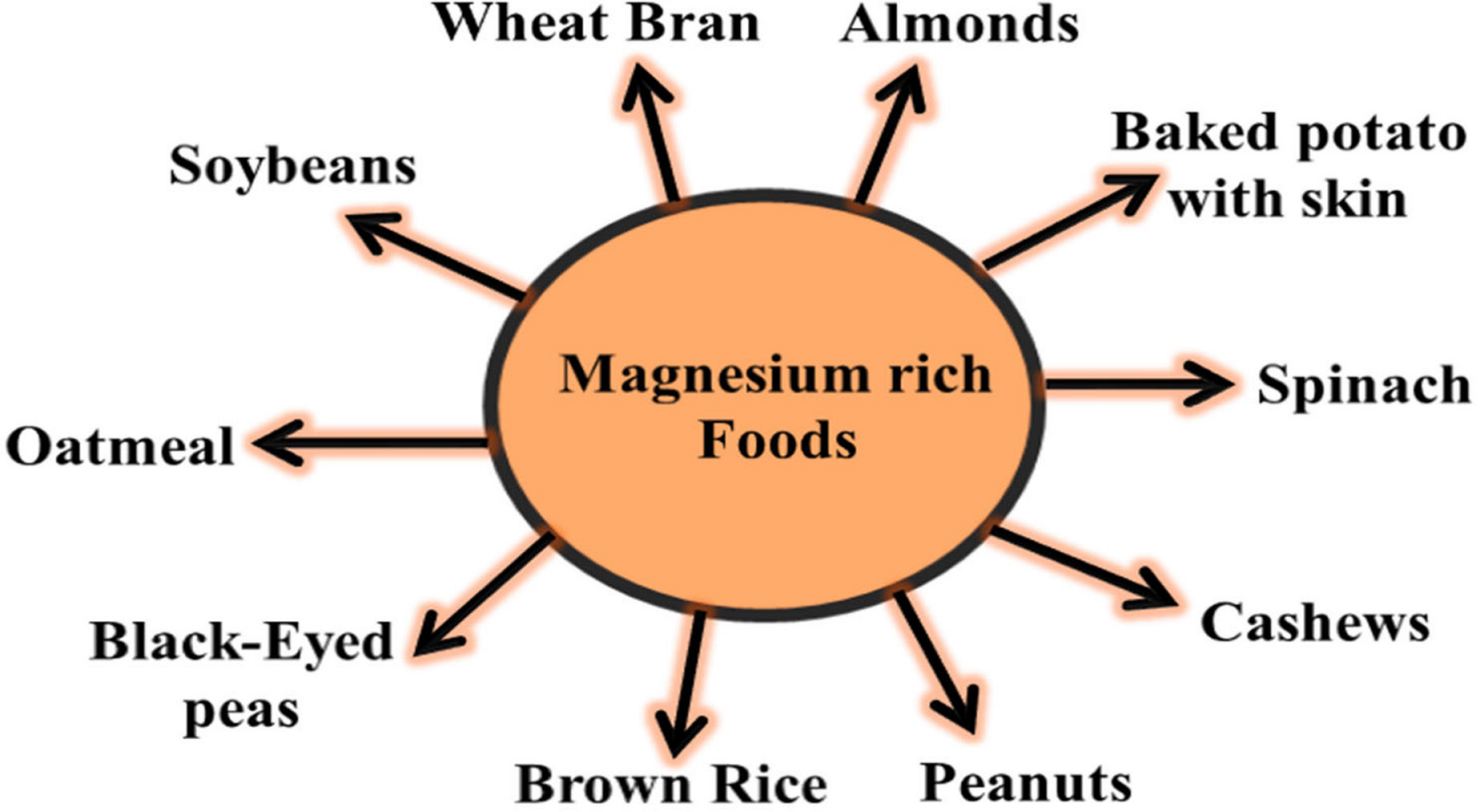
Magnesium‐rich foods.

The aim of this review is to delve into the intricate interplay between magnesium and other essential micronutrients in the context of maternal–fetal health as well as to understand the synergistic effect of magnesium with nutrients like calcium, vitamin D, potassium, zinc, iron, and vitamin B6 to optimize outcomes during pregnancy. By elucidating these interactions, we aspire to provide insights that can inform prenatal care practices, underscoring the importance of maintaining adequate magnesium levels through a balanced diet and appropriate supplementation, when necessary, all under the guidance of healthcare professionals. Figure 1 explains magnesium‐rich foods.

## 2 SIGNIFICANCE OF MATERNAL–FETAL HEALTH

Maternal–fetal health is a subject of paramount importance in healthcare, encompassing the well‐being of both the pregnant woman and her developing fetus throughout the entire pregnancy journey. It is a field of study and practice deeply rooted in the recognition that the health of the mother and the health of the fetus are intricately connected, with profound implications for both short‐term outcomes, such as a safe and healthy childbirth, and long‐term consequences, including the lifelong well‐being of the child.

### 2.1 Historical perspective

Throughout history, maternal–fetal health has been a concern, but our understanding of it has evolved significantly. In earlier times, maternal mortality during childbirth was tragically high, and the health of the fetus was often secondary to the survival of the mother. With advances in medical knowledge and healthcare practices, there has been a gradual shift toward a more comprehensive and proactive approach to maternal–fetal health (Joseph et al., 2015).

### 2.2 The interdependence between maternal and fetal health

The profound interdependence between maternal and fetal health is a cornerstone of this field. Compromised maternal health can lead to a cascade of adverse outcomes for the developing fetus. For example, maternal nutritional deficiencies, chronic illnesses, or exposure to harmful substances can have lasting effects on the fetal development process. Conversely, fetal health can also influence maternal well‐being, with conditions such as gestational diabetes or preeclampsia impacting maternal health during and after pregnancy (Monika et al., 2023).

### 2.3 Short‐term and long‐term implications

The significance of maternal–fetal health goes beyond ensuring a safe childbirth. It has substantial short‐term implications, including the prevention of preterm birth, low birth weight, and birth defects. Furthermore, maternal–fetal health has profound long‐term consequences. It is now well established that adverse conditions during fetal development, such as malnutrition or exposure to toxins, can increase the risk of chronic diseases later in life, including cardiovascular disease, diabetes, and neurodevelopmental disorders (Gluckman et al., 2008).

### 2.4 Global health impact

Maternal–fetal health is a global concern. Disparities in healthcare access and quality mean that not all pregnant women receive the same level of care, leading to disparities in maternal and fetal outcomes. This issue highlights the global significance of research and interventions aimed at improving maternal–fetal health (World Health Organization, 2021).

Hence, maternal–fetal health is a field of paramount importance with a rich historical context. It revolves around the recognition that the health of the pregnant woman and her developing fetus is deeply interconnected, with far‐reaching implications for short‐term outcomes during pregnancy and long‐term consequences that extend into the child's life. Addressing the complex interplay between maternal and fetal health is not only crucial for the well‐being of individuals but also for advancing public health on a global scale.

### 2.5 Role of micronutrients in pregnancy outcomes

Pregnancy is a dynamic physiological state where the nutritional needs of both the mother and the developing fetus increase significantly. Micronutrients, including vitamins and minerals, play a vital role in ensuring positive pregnancy outcomes. This article explores the specific contributions of various micronutrients to pregnancy and fetal health, highlighting their significance and providing relevant citations and references.

#### 2.5.1 Folic acid (vitamin B9)

Folic acid is crucial in early pregnancy as it aids in neural tube development. A deficiency can lead to neural tube defects (NTDs) in the fetus, such as spina bifida. Adequate folic acid intake is recommended, even before conception to reduce the risk of these birth defects (Greenberg et al., 2011).

#### 2.5.2 Iron

Iron is essential for preventing anemia during pregnancy, which can lead to fatigue, weakness, and other complications. Iron‐deficiency anemia can also affect fetal growth and development. Adequate iron intake, especially in the second and third trimesters, is vital (ACOG Committee Opinion No. 767, 2019).

#### 2.5.3 Calcium and vitamin D

Calcium is essential for fetal bone development, and vitamin D is necessary for calcium absorption. Insufficient calcium and vitamin D intake can lead to issues like preeclampsia and impaired fetal bone mineralization (Parvez et al., 2022).

#### 2.5.4 Vitamin C

Vitamin C contributes to collagen formation, which is vital for the development of the fetal skeleton, skin, and connective tissues. It also enhances iron absorption, aiding in the prevention of anemia (ACOG Committee Opinion No. 767, 2019).

#### 2.5.5 Zinc

Zinc is involved in numerous enzymatic processes and plays a role in immune function and DNA synthesis. Inadequate zinc intake during pregnancy can lead to growth retardation and congenital malformations in the fetus (Singh, Rastogi, et al., 2022).

#### 2.5.6 Iodine

Iodine is essential for thyroid hormone production, which is critical for fetal brain development. Severe iodine deficiency during pregnancy can result in intellectual disabilities and developmental delays in the child (Zimmermann, 2009).

#### 2.5.7 Vitamin A

Vitamin A is important for vision, immune function, and organ development. However, excessive vitamin A intake can be harmful during pregnancy, leading to birth defects, so it is essential to strike a balance (ACOG Committee Opinion No. 586, 2014).

#### 2.5.8 Omega‐3 fatty acids

Omega‐3 fatty acids, particularly docosahexaenoic acid (DHA), play a role in the development of the fetal nervous system. They also help prevent preterm birth and support healthy birth weights (Makrides et al., 2018).

#### 2.5.9 B vitamins (B6 and B12)

These vitamins are involved in amino acid metabolism and DNA synthesis. Inadequate intake can lead to neural tube defects and developmental issues (Obeid et al., 2013).

#### 2.5.10 Magnesium

Magnesium, while often overshadowed by other micronutrients, is essential for muscle relaxation, including uterine muscles. It contributes to preventing preterm contractions and complications during pregnancy (Table 1) (Makrides et al., 2014).

**TABLE 1:** Magnesium's interactions with other micronutrients.

Rationale for exploration	Importance of magnesium
Complex nutritional needs during pregnancy	Pregnancy increases nutritional demands for both mother and fetus (ACOG Committee Opinion No. 650, 2015) Micronutrients are essential for fetal growth and development (ACOG Committee Opinion (Monika et al., 2023)
Magnesium's multifaceted role	Magnesium plays a critical role in muscle relaxation, bone development, and blood pressure regulation during pregnancy (Makrides et al., 2014) Magnesium deficiency can lead to complications, such as gestational diabetes, preeclampsia, preterm birth, and low birth weight (Hromi‐Fiedler et al., 2019)
Interactions enhancing nutrient utilization	Magnesium interacts synergistically with micronutrients like calcium, vitamin D, and potassium to maintain bone health and muscle function (ACOG Committee Opinion No. 650, 2015) These interactions can enhance nutrient absorption and utilization, contributing to maternal–fetal health (Paarlberg et al., 2015)
Potential nutrient deficiencies	Pregnant women may struggle to meet increased nutritional requirements due to factors like morning sickness and dietary preferences (Hromi‐Fiedler et al., 2019) Suboptimal intake of essential micronutrients, including magnesium, can occur (Hromi‐Fiedler et al., 2019)
Implications for prenatal care	Healthcare providers guide pregnant women in maintaining optimal nutrition (Monika et al., 2023) Understanding micronutrient interactions can inform tailored dietary recommendations and supplementation strategies for improved prenatal care
Research and clinical implications	Further research into micronutrient interactions during pregnancy provides evidence‐based guidelines for healthcare professionals (Makrides et al., 2014) Identifying specific interactions and their impacts on maternal–fetal health can lead to more targeted interventions and improved outcomes

Inadequate intake of these micronutrients during pregnancy can lead to adverse outcomes, including birth defects, preterm birth, low birth weight, and maternal complications. Therefore, achieving an optimal balance of micronutrients through a well‐balanced diet and, when necessary, supplements is crucial for ensuring healthy pregnancy outcomes.

## 3 OVERVIEW OF KEY MICRONUTRIENTS CRITICAL DURING PREGNANCY

**FIGURE 2:**
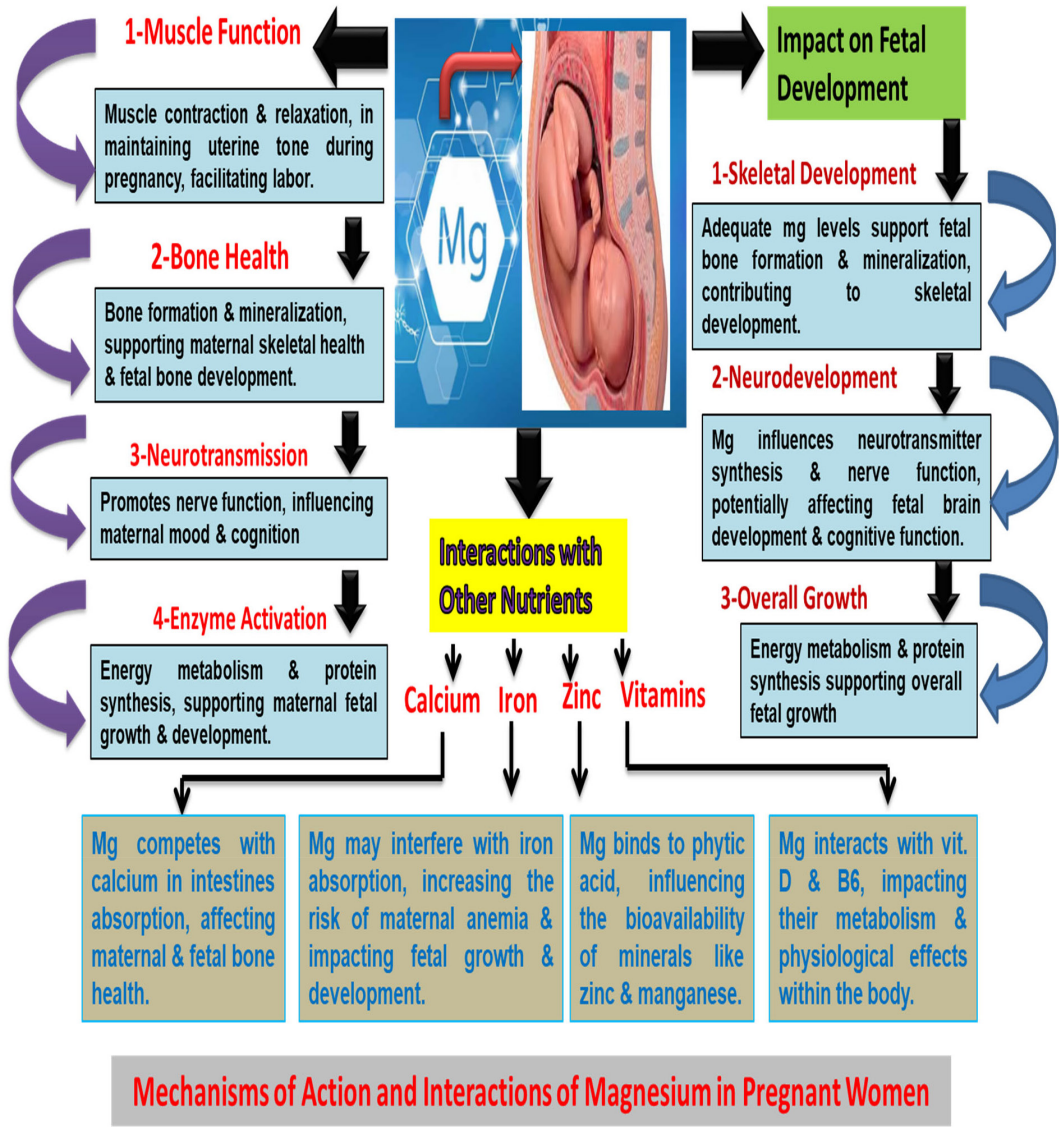
Explaining mechanisms of action and interactions of magnesium.

Micronutrients are essential for maternal and fetal health (Table 2). These micronutrients impact maternal health (Figure 2). Key micronutrients critical during pregnancy include folate, which is essential for DNA synthesis and preventing neural tube defects; iron, which is necessary for hemoglobin production and preventing anemia; calcium and vitamin D, vital for fetal bone development; vitamin C, important for collagen synthesis and iron absorption; zinc, involved in enzymatic processes and immune function; iodine, crucial for fetal brain development and growth; vitamin A, essential for vision and immune function; and omega‐3 fatty acids, particularly DHA, critical for fetal nervous system development and preventing preterm birth (ACOG Committee Opinion No. 586, 2014; ACOG Committee Opinion No. 767, 2019; ACOG Committee Opinion No. 795, 2019; Makrides et al., 2018; Parvez et al., 2021; Singh et al., 2021; Singh, Rastogi, et al., 2022; Zimmermann, 2009) (Table 3). These micronutrients are essential for ensuring a healthy pregnancy and optimal fetal development, as they are required in the development of bones and other vital organs in the body.

**TABLE 2:** Micronutrients essential for maternal–fetal health.

Micronutrient	Function
Folate (vitamin B9)	Essential for DNA synthesis and cell division, preventing neural tube defects (NTDs) (ACOG Committee Opinion No. 795, 2019)
Iron	Necessary for hemoglobin production, preventing iron‐deficiency anemia in both mother and baby (ACOG Committee Opinion No. 767, 2019)
Calcium and vitamin D	Calcium is crucial for fetal bone and teeth development; vitamin D aids in calcium absorption (Parvez et al., 2021)
Vitamin C	Essential for collagen synthesis, promoting healthy skin, blood vessels, and connective tissues (Singh et al., 2021)
Zinc	Involved in enzymatic processes, supports immune function, and aids in DNA synthesis; essential for fetal growth (Singh, Rastogi, et al., 2022)
Iodine	Crucial for thyroid hormone production, essential for fetal brain development and overall growth (Zimmermann, 2009)
Vitamin A	Vital for vision, immune function, and organ development; excess intake can be harmful during pregnancy (ACOG Committee Opinion No. 586, 2014)
Omega‐3 fatty acids	Critical for fetal nervous system development, helps prevent preterm birth, and supports healthy birth weights (Makrides et al., 2018)

**TABLE 3:** Roles of specific micronutrients in fetal development and maternal well‐being.

Micronutrient	Role in fetal development	Role in maternal well‐being
Folate (vitamin B9)	Crucial for neural tube formation, preventing NTDs; Supports overall fetal growth and development	Prevents anemia in pregnant women, reduces the risk of preeclampsia, and supports maternal health (ACOG Committee Opinion No. 795, 2019)
Iron	Essential for fetal hemoglobin production, ensuring oxygen supply to the fetus; Supports fetal growth and organ development	Prevents iron‐deficiency anemia in pregnant women, reducing fatigue and complications (ACOG Committee Opinion No. 767, 2019)
Calcium and vitamin D	Calcium: Critical for fetal bone and teeth formation; Vitamin D: Aids in calcium absorption, supporting fetal skeletal development	Calcium: Prevents maternal bone loss; Vitamin D: Supports bone health and immune function (Parvez et al., 2021)
Vitamin C	Important for collagen synthesis, contributing to fetal skin, blood vessels, and connective tissues	Enhances iron absorption, prevents anemia in pregnant women, and supports the immune system (Singh et al., 2021)
Zinc	Involved in enzymatic processes essential for fetal growth, DNA synthesis, and cellular functioning	Supports maternal immune function, wound healing, and overall health during pregnancy (Singh, Rastogi, et al., 2022)
Iodine	Crucial for thyroid hormone production, essential for fetal brain development, nervous system functioning, and overall growth	Supports maternal thyroid health, ensuring stable energy levels and overall well‐being (Zimmermann, 2009)

## 4 MAGNESIUM'S BIOLOGICAL FUNCTIONS

Magnesium is an essential mineral that plays numerous critical roles in the human body. Its functions extend to various physiological processes, highlighting its significance for overall health and well‐being (Figure 2). Magnesium acts as a cofactor for hundreds of enzymes involved in various metabolic reactions, including ATP (adenosine triphosphate) production, which is the primary energy currency of cells (Swaminathan, 2003). Additionally, magnesium is crucial for muscle function and relaxation, regulating neuromuscular signals and the contraction and relaxation of muscles, including the heart (Rude et al., 1998). Furthermore, it plays a vital role in the nervous system by participating in the regulation of neurotransmitter release and receptor function, contributing to proper nerve function and signaling (DiNicolantonio et al., 2020). These diverse functions emphasize the essential nature of magnesium for maintaining optimal health (Table 4).

**TABLE 4:** Roles of magnesium in the body.

Function	Description
Enzyme activation and ATP production	Magnesium acts as a cofactor for numerous enzymes involved in metabolic reactions, including ATP production, the primary energy currency of cells (Swaminathan, 2003)
Muscle function and contraction	Magnesium is essential for regulating neuromuscular signals and the contraction and relaxation of muscles, including the heart (Rude et al., 1998)
Nervous system function	Magnesium participates in the regulation of neurotransmitter release and receptor function, contributing to proper nerve function and signaling (DiNicolantonio et al., 2020)
Bone health	Magnesium plays a role in bone mineralization and bone density maintenance, influencing overall skeletal health (Castiglioni & Maier, 2011)
Cardiovascular health	Magnesium supports the normal function of heart muscles, helps regulate blood pressure, and is associated with a reduced risk of cardiovascular diseases (Del Gobbo et al., 2013)
Blood glucose regulation	Magnesium is involved in insulin release and action, contributing to the regulation of blood glucose levels (Barbagallo et al., 1997)
Immune system support	Magnesium is necessary for immune system function, influencing immune cell activity and cytokine production (Razzaque, 2018)

### 4.1 Implications of magnesium deficiency during pregnancy

Magnesium deficiency during pregnancy can have significant implications for both maternal health and fetal development. This essential mineral plays a vital role in numerous physiological processes, and its insufficiency can lead to various complications. For the mother, magnesium deficiency may increase the risk of developing conditions, such as gestational diabetes, preeclampsia, and hypertension (Diaz‐Castro et al., 2016). Preeclampsia, in particular, is a serious condition characterized by high blood pressure and organ damage, and it poses risks to both the mother and the fetus (Ferguson, 2018). Additionally, magnesium deficiency can contribute to muscle cramps, fatigue, and mood disturbances, which can negatively impact the overall well‐being of pregnant women (Kass et al., 2019). In terms of fetal development, inadequate magnesium intake has been associated with adverse outcomes, including preterm birth, low birth weight, and even developmental abnormalities (Institute of Medicine (US) Standing Committee on the Scientific Evaluation of Dietary Reference Intakes and its Panel on Folate, Other B Vitamins, and Choline, 1998). Magnesium is essential for proper bone development, and its deficiency may impair the fetal skeletal system. Furthermore, magnesium is involved in regulating neurotransmitter release and neuronal function, and its shortage could affect the developing nervous system of the fetus (Zhang et al., 2019). To mitigate these risks, it is crucial for pregnant women to ensure they meet their daily magnesium requirements, either through dietary sources or through supplements prescribed by healthcare providers. Proper prenatal care and monitoring of magnesium levels can help prevent these potential complications and support both maternal and fetal health (Table 5).

**TABLE 5:** Interactions between magnesium and other micronutrients.

Micronutrient	Interaction with magnesium
Calcium	Magnesium enhances calcium absorption in the intestines and supports its utilization in bones (Rude et al., 2009a)
Vitamin D	Magnesium is necessary for the activation of vitamin D in the body; Facilitates calcium absorption, supporting bone health and overall mineral balance (Uwitonze & Razzaque, 2018)
Potassium	Magnesium and potassium work together to maintain electrolyte balance; Crucial for muscle and nerve function, as well as overall fluid balance (Ter Braake et al., 2020)
Zinc	Magnesium enhances zinc absorption in the intestines and supports its activation; Supports immune function, cellular growth, and DNA synthesis (Sturniolo et al., 2001)
Iron	Magnesium regulates iron metabolism, enhancing non‐heme iron absorption; Essential for preventing iron deficiency and supporting various physiological processes (Hunt, 2003)
Vitamin B6	Magnesium is required for the activation of vitamin B6 in the body; Crucial for amino acid metabolism and neurotransmitter synthesis (Tam et al., 2003)

### 4.2 Calcium and magnesium: Balance for bone health and more

Calcium and magnesium are essential minerals that work in harmony within the body to support various physiological processes, with a primary focus on bone health. Their balanced interplay is critical for maintaining strong bones and preventing osteoporosis. Calcium is renowned for its role in bone mineralization, providing the structural integrity of bones, while magnesium plays a complementary role by ensuring proper calcium utilization. Calcium, primarily stored in bones, is released into the bloodstream when needed for vital functions, such as muscle contraction, nerve transmission, and blood clotting. Magnesium is integral to this process as it facilitates calcium's entry into cells and its activation within the body. Without sufficient magnesium, calcium may accumulate in soft tissues, potentially leading to calcification and contributing to conditions like kidney stones (Rude et al., 2009a).

Furthermore, magnesium regulates parathyroid hormone (PTH) secretion, which in turn influences calcium homeostasis. PTH stimulates the release of calcium from bones when levels are low and enhances renal reabsorption of calcium. Magnesium's involvement in this hormonal control system is crucial for maintaining the delicate balance of calcium in the body. Beyond bone health, this dynamic duo has far‐reaching effects on overall well‐being. Adequate calcium intake, with magnesium support, is associated with the prevention of hypertension and cardiovascular diseases. Magnesium, in its own right, contributes to muscle relaxation, reducing the risk of muscle cramps and spasms. Additionally, magnesium supports the nervous system, promoting mental relaxation and stress reduction. It is worth noting that achieving the ideal calcium–magnesium balance can be challenging, as many individuals consume excessive calcium while falling short on magnesium intake. The recommended dietary ratio of calcium to magnesium is approximately 2:1. However, modern diets often skew this balance, with a higher emphasis on calcium‐rich dairy products and supplements (DiNicolantonio et al., 2020; Ter Braake et al., 2020).

In conclusion, calcium and magnesium form a dynamic duo that promotes bone health and overall well‐being. Their intricate interplay goes beyond bone mineralization and extends to cardiovascular health, muscle function, and nervous system regulation. Striking the right balance between these minerals through a balanced diet and appropriate supplementation when necessary is essential for maintaining optimal health.

### 4.3 Magnesium and zinc: Insights into immune function and growth

Magnesium and zinc are indispensable micronutrients that play pivotal roles in supporting immune function and growth, highlighting their critical significance for overall health. Magnesium is involved in various aspects of immune response. It regulates immune cell activity, influences the production of cytokines and antibodies, and supports the body's defense mechanisms against infections (Razzaque, 2018). Zinc, on the other hand, is a key player in immune function. It is essential for the development and functioning of immune cells, including T cells and natural killer cells, and it contributes to the body's defense against pathogens (Wessels et al., 2017). The interaction between magnesium and zinc is particularly noteworthy. Magnesium enhances the absorption of zinc in the intestines, ensuring that an adequate amount of this essential mineral is available for immune processes and overall growth (Sturniolo et al., 2001). Zinc, in turn, supports cellular growth, DNA synthesis, and wound healing. It is especially vital for the rapid growth and development that occurs during pregnancy and childhood. Furthermore, both magnesium and zinc are involved in anti‐inflammatory processes. Magnesium helps regulate inflammation by modulating the activity of inflammatory cytokines (Tam et al., 2003). Zinc, as an antioxidant, combats oxidative stress and inflammation, contributing to overall immune system balance. Inadequate levels of either magnesium or zinc can compromise immune function and growth. Magnesium deficiency has been associated with a weakened immune response, making individuals more susceptible to infections (Razzaque, 2018). Zinc deficiency, similarly, can impair immune function, leading to increased vulnerability to illnesses and growth retardation, especially in children (Wessels et al., 2017). To ensure robust immune function and support growth, it is crucial to maintain optimal levels of both magnesium and zinc through a balanced diet or supplements when necessary. Their synergistic interactions underscore the importance of these micronutrients in fortifying the body's immune defenses and promoting healthy growth and development.

### 4.4 Magnesium and iron: Iron absorption and anemia considerations

The relationship between magnesium and iron is crucial, especially concerning iron absorption and its implications for preventing anemia. Iron is an essential mineral involved in the production of hemoglobin, which carries oxygen in the blood. Adequate iron intake is essential to prevent anemia, a condition characterized by a deficiency of red blood cells or hemoglobin, resulting in fatigue, weakness, and other health complications. Magnesium plays a pivotal role in this context. It is required for the proper functioning of the enzyme responsible for the conversion of dietary iron into a form that can be absorbed by the intestines, known as the ferrous (Fe2+) form (Hurrell & Egli, 2010). In the absence of sufficient magnesium, the conversion of iron to its absorbable form may be compromised, leading to decreased iron absorption. This can contribute to the development of iron‐deficiency anemia. Furthermore, magnesium helps regulate iron metabolism through its interaction with various proteins involved in iron transport and storage (Blache et al., 2020). It ensures that iron is distributed appropriately throughout the body and supports its utilization in essential physiological processes. Anemia, particularly during pregnancy, can have severe consequences for both the mother and the developing fetus. Iron‐deficiency anemia in pregnant women has been associated with preterm birth, low birth weight, and developmental abnormalities in the fetus (Al‐Awaida et al., 2020). Therefore, maintaining adequate levels of both magnesium and iron is of paramount importance, especially during pregnancy, to prevent anemia and support overall health. In conclusion, magnesium's role in iron absorption and iron metabolism is critical for preventing iron‐deficiency anemia. Ensuring sufficient magnesium intake through a balanced diet or supplements, when recommended by healthcare professionals, can help optimize iron utilization, supporting overall health and well‐being.

### 4.5 Magnesium and vitamin D: Calcium homeostasis and beyond

The dynamic interplay between magnesium and vitamin D extends far beyond their individual roles, primarily concerning calcium homeostasis and overall health. While vitamin D is essential for calcium absorption and utilization, magnesium is the unsung hero that ensures this process operates optimally. Vitamin D, upon activation in the skin and further conversion in the liver and kidneys, facilitates the absorption of calcium in the intestines. However, magnesium is a critical cofactor in this activation process, ensuring that vitamin D is metabolized to its active form, calcitriol (Uwitonze & Razzaque, 2018). Calcitriol, in turn, supports calcium absorption from the diet, maintaining adequate calcium levels in the bloodstream. Beyond calcium homeostasis, both magnesium and vitamin D exert various health benefits. Magnesium supports muscle relaxation, nerve function, and cardiovascular health (DiNicolantonio et al., 2020). Vitamin D influences immune function, hormonal regulation, and bone health. Additionally, both nutrients have anti‐inflammatory properties and play roles in mental well‐being.

Adequate levels of magnesium and vitamin D are essential for overall health, and their deficiency can lead to various complications. Magnesium deficiency can impair vitamin D metabolism, potentially leading to suboptimal calcium absorption and utilization (DiNicolantonio et al., 2020). Conversely, vitamin D deficiency can hinder magnesium absorption in the intestines, creating a feedback loop that exacerbates deficiencies in both nutrients. This intricate relationship underscores the importance of maintaining balanced levels of magnesium and vitamin D through a well‐rounded diet or supplements, when advised by healthcare professionals. Achieving the proper equilibrium between these nutrients ensures optimal calcium homeostasis, promotes overall health, and reduces the risk of associated health conditions.

## 5 SYNERGISTIC EFFECTS ON MATERNAL HEALTH

Maternal health is a multifaceted concern, and the synergistic effects of essential micronutrients can have a profound impact on the well‐being of pregnant women. During pregnancy, the body undergoes significant physiological changes, and the demand for various nutrients increases substantially. The interactions and synergies between these nutrients play a crucial role in ensuring maternal health.

- *Calcium and vitamin D*: Calcium is vital for fetal skeletal development, while vitamin D enhances calcium absorption. Together, they help prevent maternal osteoporosis and support the growing baby's bone health (Prentice, 2003).
- *Iron and vitamin C*: Iron is essential for preventing anemia during pregnancy, and vitamin C enhances iron absorption. This synergy aids in maintaining optimal hemoglobin levels, reducing the risk of maternal anemia (Milman, 2012).
- *Folate and vitamin B12*: Folate is crucial for preventing neural tube defects in the fetus, and vitamin B12 is needed for folate activation. Their synergy is vital for fetal brain and spinal cord development while also benefiting maternal nerve health (Green et al., 2017).
- *Magnesium and calcium*: Magnesium aids in calcium absorption and supports muscle relaxation. This synergy helps prevent muscle cramps and complications related to muscle spasms during pregnancy (DiNicolantonio et al., 2020).
- *Zinc and copper*: Zinc is necessary for fetal growth, while copper helps regulate zinc metabolism. Their synergy contributes to optimal fetal development and supports maternal immunity (Cordano et al., 2016).
- *Omega‐3 fatty acids and vitamin D*: Omega‐3 fatty acids are essential for brain development in the fetus, and vitamin D enhances their utilization. Together, they promote healthy neural development and immune function in both the mother and the baby (Suresh & Das, 2019).
- *Vitamin A and zinc*: Vitamin A supports vision and immune function, while zinc helps maintain vitamin A levels. Their synergy is essential for maternal eye health and immune system support (Shankar et al., 2000).

## 6 HOW MAGNESIUM INTERACTIONS IMPACT MATERNAL WELL‐BEING

Magnesium, through its intricate interactions with various micronutrients, exerts a profound influence on maternal well‐being during pregnancy. These interactions are vital for ensuring optimal health and mitigating potential complications that can arise during this critical period.

- *Calcium and magnesium*: The balanced interplay between calcium and magnesium is essential for maternal health. Magnesium facilitates calcium absorption and utilization, preventing muscle cramps and spasms that are common during pregnancy (DiNicolantonio et al., 2020). This synergy contributes to overall muscle and nerve function, reducing discomfort for expectant mothers.
- *Vitamin D and magnesium*: Vitamin D, critical for calcium absorption, relies on magnesium for its activation (Uwitonze & Razzaque, 2018). Magnesium ensures that the body efficiently utilizes vitamin D, contributing to optimal calcium metabolism. This synergy supports maternal bone health, a vital consideration during pregnancy.
- *Iron and magnesium*: Iron is crucial for preventing anemia during pregnancy, while magnesium regulates iron metabolism and enhances its absorption (Blache et al., 2020). This interaction supports maternal hemoglobin levels, reducing the risk of iron‐deficiency anemia, which can lead to fatigue and other complications.
- *Zinc and magnesium*: Magnesium enhances zinc absorption in the intestines (Sturniolo et al., 2001). This interaction supports maternal immune function, cellular growth, and DNA synthesis. An adequately functioning immune system is vital during pregnancy to protect both the mother and the developing fetus.
- *Vitamin B6 and magnesium*: Vitamin B6 is crucial for amino acid metabolism and neurotransmitter synthesis, and magnesium aids in its activation (Tam et al., 2003). This interaction contributes to maternal mental well‐being by supporting neurotransmitter function, potentially reducing the risk of mood disturbances.

## 7 COMBINED EFFECTS OF MAGNESIUM AND OTHER MICRONUTRIENTS ON GESTATIONAL DIABETES MELLITUS RISK

Gestational diabetes mellitus (GDM) is a significant concern during pregnancy, affecting both maternal and fetal health. Emerging research suggests that the combined effects of magnesium with other micronutrients can influence the risk of developing GDM.

- *Magnesium and calcium*: The interaction between magnesium and calcium is vital in maintaining glucose homeostasis. Magnesium enhances insulin sensitivity, while calcium, when balanced with magnesium, can reduce the risk of GDM. A study found that women with higher magnesium‐to‐calcium ratios had a reduced risk of GDM (Zhang et al., 2016).
- *Magnesium and zinc*: Zinc supports insulin function and pancreatic beta‐cell activity, and magnesium facilitates zinc absorption (Vazquez‐Lorente et al., 2019). Combined, they may contribute to improve glucose metabolism and a reduced risk of GDM.
- *Magnesium and vitamin D*: Vitamin D plays a role in insulin sensitivity, and magnesium is necessary for vitamin D activation (Uwitonze & Razzaque, 2018). This synergy may support better glycemic control during pregnancy.
- *Magnesium and iron*: Iron is essential for preventing anemia during pregnancy, and magnesium aids in iron metabolism. Maintaining proper iron levels can potentially reduce GDM risk, as anemia has been associated with GDM (Zhang et al., 2021).

## 8 POTENTIAL REDUCTION IN PREGNANCY‐INDUCED HYPERTENSION THROUGH SYNERGIES

Pregnancy‐induced hypertension, including conditions like preeclampsia and gestational hypertension, poses significant risks to maternal and fetal health. Recent research has shed light on the potential reduction in the risk of these conditions through synergies between key micronutrients.

- *Calcium and magnesium*: A balanced intake of calcium and magnesium may reduce the risk of pregnancy‐induced hypertension. Calcium supports blood vessel relaxation and helps maintain healthy blood pressure levels, while magnesium enhances this effect (Hofmeyr et al., 2010). Studies suggest that combined supplementation of calcium and magnesium may be associated with a lower incidence of preeclampsia (Hofmeyr et al., 2010).
- *Magnesium and potassium*: Magnesium and potassium work together to maintain electrolyte balance and proper muscle function. This synergy can help prevent hypertension during pregnancy, as imbalances in electrolytes can contribute to high blood pressure (Ter Braake et al., 2020).
- *Magnesium and zinc*: Both magnesium and zinc play roles in vascular health and immune function. Their combined effects may contribute to reducing inflammation and oxidative stress, which are associated with pregnancy‐induced hypertension (Tam et al., 2003).
- *Vitamin D and magnesium*: Vitamin D, activated with the help of magnesium, may have a protective effect against preeclampsia (Uwitonze & Razzaque, 2018). Adequate vitamin D levels are associated with a reduced risk of hypertensive disorders during pregnancy (Wei et al., 2017).
- *Omega‐3 fatty acids and magnesium*: Omega‐3 fatty acids have anti‐inflammatory properties and support cardiovascular health. When combined with magnesium, they may help reduce the risk of pregnancy‐induced hypertension by promoting healthy blood vessel function (Gao et al., 2016).

## 9 FETAL DEVELOPMENT AND MICRONUTRIENT INTERPLAYS

Fetal development is a complex and highly regulated process that depends on a myriad of micronutrients for optimal outcomes. Micronutrient interactions play a critical role in shaping the growth and health of the developing fetus.

### 9.1 The interplay between nutrition and stress in pregnancy

- *Folate and vitamin B12*: Folate, in conjunction with vitamin B12, is vital for neural tube development and the formation of the nervous system (Green et al., 2017). Deficiencies in either nutrient can lead to neural tube defects, emphasizing the importance of their interplay during pregnancy.
- *Calcium and magnesium*: Calcium supports fetal bone and teeth development, and magnesium ensures proper calcium utilization (DiNicolantonio et al., 2020). The balanced interplay between these minerals is essential for healthy skeletal growth in the fetus.
- *Iron and vitamin C*: Iron is crucial for fetal growth, and vitamin C enhances iron absorption (Milman, 2012). Their interplay is particularly significant in preventing iron‐deficiency anemia, which can hinder fetal development.
- *Zinc and copper*: Zinc is essential for cellular growth and DNA synthesis in the fetus, while copper helps regulate zinc metabolism (Cordano et al., 2016). Their synergistic effects contribute to optimal fetal development.
- *Omega‐3 fatty acids and vitamin D*: Omega‐3 fatty acids support brain and visual development in the fetus, and vitamin D enhances their utilization (Suresh & Das, 2019). This interplay is crucial for promoting healthy neural development.
- *Vitamin A and zinc*: Vitamin A is essential for vision and immune function in the fetus, and zinc helps maintain vitamin A levels (Shankar et al., 2000). Their interplay ensures optimal fetal eye health and immune system support.
- *Vitamin D and magnesium*: Magnesium assists in vitamin D activation, which plays a role in fetal bone health (Uwitonze & Razzaque, 2018). This interplay contributes to the development of a strong skeletal system.

### 9.2 Magnesium's contribution to neural development and brain health

Magnesium, often referred to as the “brain mineral,” plays a pivotal role in neural development and overall brain health. Its involvement in various physiological processes is essential for shaping the developing fetal brain and maintaining cognitive function throughout life.

- *Neural tube development*: Magnesium is crucial during the early stages of pregnancy for neural tube development. It works in tandem with other micronutrients, such as folate and vitamin B12, to ensure the proper closure of the neural tube, a critical structure that eventually forms the brain and spinal cord (Green et al., 2017).
- *Neurotransmitter regulation*: Magnesium participates in the regulation of neurotransmitters, the chemical messengers of the nervous system. It is involved in the synthesis and release of neurotransmitters like serotonin and dopamine, which play key roles in mood, cognition, and overall brain function (Boyle et al., 2017).
- *Nervous system function*: Magnesium supports the function of the nervous system by facilitating nerve impulse transmission and muscle contraction. It is essential for maintaining neuromuscular integrity, which includes coordination and motor skills (DiNicolantonio et al., 2020).
- *Brain protection*: Magnesium has neuroprotective properties. It helps reduce the risk of neuroinflammation and oxidative stress, which are associated with various neurological disorders, including Alzheimer's disease and Parkinson's disease (Sartori et al., 2012).
- *Memory and learning*: Adequate magnesium levels have been linked to improved memory and learning capabilities. Magnesium supports synaptic plasticity, a mechanism underlying learning and memory formation (Slutsky et al., 2010).

### 9.3 The combined roles of magnesium and other micronutrients in preventing birth defects

Birth defects can have lifelong consequences, making prevention a top priority during pregnancy. Magnesium, in combination with other essential micronutrients, plays a crucial role in reducing the risk of birth defects through various mechanisms.

- *Folate and vitamin B12*: Folate, in synergy with vitamin B12, is essential for preventing neural tube defects (NTDs) in the developing fetus (Green et al., 2017). These nutrients work together to support DNA synthesis and methylation processes required for proper neural tube closure.
- *Calcium and magnesium*: Adequate calcium and magnesium intake during pregnancy is vital for fetal skeletal development and the prevention of skeletal deformities (DiNicolantonio et al., 2020). Magnesium aids in calcium utilization, ensuring the structural integrity of the developing bones.
- *Iron and vitamin C*: Iron deficiency during pregnancy can lead to anemia and increase the risk of birth defects (Milman, 2012). Vitamin C enhances iron absorption, making it crucial for maintaining optimal iron levels necessary for fetal development.
- *Zinc and copper*: Zinc plays a role in preventing developmental abnormalities, and copper helps regulate zinc metabolism (Cordano et al., 2016). Their combined effects contribute to proper fetal growth and the prevention of congenital malformations.
- *Omega‐3 fatty acids and vitamin D*: Omega‐3 fatty acids, in conjunction with vitamin D, promote healthy fetal brain and visual development (Suresh & Das, 2019). This synergy is essential for reducing the risk of neurological birth defects.
- *Vitamin A and zinc*: Vitamin A and zinc work together to support immune function and prevent congenital infections that can lead to birth defects (Shankar et al., 2000). This interplay helps safeguard against developmental abnormalities caused by infections.

### 9.4 Nutrient interactions affecting placental development and function

The placenta plays a critical role in facilitating the exchange of nutrients and oxygen between the mother and the developing fetus. Nutrient interactions within the maternal body significantly impact placental development and function, ultimately influencing fetal growth and health.

- *Folate and vitamin B12*: Folate and vitamin B12 interact to support DNA synthesis and methylation processes, which are essential for placental development (Green et al., 2017). Deficiencies in these nutrients can lead to impaired placental function, potentially affecting fetal growth.
- *Calcium and magnesium*: Adequate calcium and magnesium levels are crucial for placental development and function. Magnesium aids in calcium absorption and utilization, contributing to the structural integrity of the placenta (DiNicolantonio et al., 2020). An imbalance between these minerals can disrupt placental function and nutrient exchange.
- *Iron and vitamin C*: Iron deficiency during pregnancy can impair placental development and function, leading to reduced oxygen and nutrient transport to the fetus (Milman, 2012). Vitamin C enhances iron absorption, making it vital for maintaining optimal placental iron levels.
- *Zinc and copper*: Zinc and copper play roles in placental angiogenesis, the formation of new blood vessels within the placenta. Their combined effects are essential for ensuring an adequate blood supply to support fetal growth and nutrient exchange (Cordano et al., 2016).
- *Omega‐3 fatty acids and vitamin D*: Omega‐3 fatty acids and vitamin D contribute to placental vascular health. They help maintain the integrity of placental blood vessels, ensuring efficient nutrient and oxygen transfer to the fetus (Suresh & Das, 2019).
- *Vitamin A and zinc*: Vitamin A and zinc interactions influence placental immune function and protect against infections that can compromise placental health (Shankar et al., 2000). A well‐functioning placental immune system is crucial for fetal protection.

## 10 CLINICAL SIGNIFICANCE

### 10.1 Magnesium supplementation strategies during pregnancy

Magnesium supplementation during pregnancy can play a vital role in optimizing maternal and fetal health outcomes. The necessity of ensuring appropriate magnesium levels is underscored by various studies. For instance, Makrides and Crosby (2014) illuminate the importance of tailoring magnesium dosage and choosing suitable formulations to meet individual requirements, recognizing its role in overall well‐being and in the mitigation of pregnancy complications. Moreover, the strategic use of magnesium, particularly magnesium sulfate, in high‐risk pregnancies has been noted to confer neuroprotective benefits to the fetus. Doyle et al. (2009) highlighted that magnesium sulfate administration in women at risk of preterm birth serves to minimize the potential occurrence of cerebral palsy and safeguard fetal neurodevelopment. In relation to preeclampsia, a condition characterized by hypertension and organ damage in pregnant women, magnesium sulfate is employed as a critical intervention. Duley et al. (2010) detailed how the prophylactic administration of magnesium sulfate in preeclampsia cases serves to manage and prevent seizures, representing a crucial strategy in navigating the risks of this condition and ensuring safety for both mother and child. Thus, the strategic employment of magnesium supplementation, whether for general health or for targeted interventions, aligns with a model of care that prioritizes optimal outcomes in maternal–fetal health, thereby underscoring its pivotal role in prenatal care. It is imperative to note that any supplementation strategy should always be implemented under the guidance and supervision of healthcare professionals to ensure safety and efficacy.

### 10.2 Monitoring micronutrient status and addressing deficiencies

Monitoring micronutrient status and managing deficiencies, particularly during pregnancy, is crucial due to the pronounced effects on both maternal and fetal health outcomes. A comprehensive assessment of nutritional status helps in identifying and addressing potential micronutrient deficiencies. Allen (2000) (Singh, Fedacko, et al., 2022) underscores the importance of preventing deficiencies like anemia, elaborating on its consequences, including preterm delivery and perinatal mortality, which delineates the vital role of micronutrients like iron in pregnancy. By employing various strategies, such as dietary assessments, laboratory testing, and targeted supplementation, healthcare providers can proactively mitigate the risks associated with micronutrient deficiencies. Thorough and regular monitoring ensures that nutritional needs are met throughout pregnancy and that potential issues can be preemptively addressed, safeguarding the health and well‐being of both mother and fetus. Thus, structured monitoring and a nuanced understanding of micronutrient requirements during pregnancy are pivotal in nurturing optimal health outcomes. Always ensure that any intervention or supplementation is under the guidance and supervision of healthcare professionals to ensure safety and efficacy.

### 10.3 Negative aspects of the use of magnesium supplementation during pregnancy

Magnesium supplementation during pregnancy is commonly recommended due to its crucial role in maternal and fetal health. However, while magnesium offers numerous benefits, it is essential to acknowledge and understand its potential negative aspects to ensure a balanced approach to supplementation. One of the primary concerns associated with magnesium supplementation during pregnancy is the possibility of gastrointestinal discomfort. As per Shariati et al. (2016), some women may experience side effects such as diarrhea, nausea, or abdominal cramping when taking magnesium supplements. These symptoms can be particularly troublesome during pregnancy, as they may exacerbate existing discomfort or contribute to dehydration. According to Shariati et al. (2016), it is important for healthcare providers to counsel pregnant women on ways to minimize gastrointestinal side effects, such as adjusting the timing or dosage of supplementation or selecting alternative forms of magnesium that are better tolerated (Asemi et al., 2015). Excessive magnesium intake can also lead to magnesium toxicity, which poses significant risks to both the mother and the fetus. Magnesium toxicity can occur when intake surpasses the body's ability to excrete it, resulting in elevated levels of magnesium in the bloodstream. Symptoms of magnesium toxicity may include low blood pressure, irregular heartbeats, respiratory distress, and muscle weakness. In severe cases, magnesium toxicity can lead to cardiac arrest or respiratory failure, posing a serious threat to maternal and fetal well‐being. Healthcare providers must monitor pregnant women closely for signs of magnesium toxicity and adjust supplementation regimens accordingly to prevent adverse outcomes (Oster et al., 1980).

Furthermore, magnesium supplementation may interact with certain medications commonly used during pregnancy. For example, magnesium can interfere with the absorption or efficacy of antibiotics, thyroid medications, and medications used to manage gestational diabetes or hypertension. These interactions can compromise the effectiveness of treatment and potentially pose risks to maternal or fetal health. Pregnant women should inform their healthcare providers about all medications and supplements they are taking to identify and manage potential interactions effectively (NCCIH, 2017). Additionally, an imbalance in magnesium levels can impact the absorption or utilization of other essential nutrients, further complicating maternal and fetal health. Magnesium competes with calcium for absorption in the intestines, and excessive magnesium intake may disrupt the body's calcium balance. This imbalance can potentially lead to issues such as calcium deficiency, which is essential for fetal bone development and maternal bone health (Ranade & Somberg, 2001). Moreover, magnesium supplementation may interfere with the absorption of iron, zinc, and other micronutrients critical for maternal and fetal well‐being (Hess & King, 2009). Healthcare providers must consider these interactions when recommending magnesium supplementation to pregnant women and may need to adjust other aspects of their diet or supplementation regimen to ensure optimal nutrient intake.

Magnesium supplementation offers numerous benefits for maternal and fetal health during pregnancy; it is essential to recognize and address potential negative aspects as well. Gastrointestinal discomfort, magnesium toxicity, interactions with medications, and disruptions to nutrient absorption are among the key concerns associated with magnesium supplementation during pregnancy. Healthcare providers must educate pregnant women about these risks, monitor them closely for adverse effects, and tailor supplementation regimens to minimize potential harm while maximizing the benefits of magnesium for maternal and fetal well‐being. By adopting a balanced and informed approach to magnesium supplementation, healthcare providers can help ensure the safest and most effective care for pregnant women and their babies.

### 10.4 Negative impact of the interaction between magnesium and other nutrients

The interaction between magnesium and other nutrients can have both positive and negative impacts on maternal and fetal health during pregnancy. While magnesium plays a crucial role in numerous physiological processes, including energy metabolism, muscle function, and nerve transmission, its interactions with other nutrients must be carefully considered to ensure optimal maternal and fetal well‐being. One significant concern regarding the interaction between magnesium and nutrients is the potential for nutrient absorption interference. Magnesium competes with other minerals, such as calcium and iron, for absorption in the intestines. Excessive magnesium intake may disrupt the body's ability to absorb these essential nutrients, leading to deficiencies that can adversely affect maternal health and fetal development. For example, calcium is essential for fetal bone formation and maternal bone health, and magnesium's interference with calcium absorption may compromise these critical processes. Similarly, iron deficiency can lead to maternal anemia and increase the risk of preterm birth and low birth weight in infants, highlighting the importance of maintaining optimal iron levels during pregnancy (de Baaij et al., 2015). Moreover, magnesium supplementation may affect the utilization of other nutrients within the body. For instance, magnesium is involved in the activation of vitamin D, which is essential for calcium absorption and bone health. However, excessive magnesium intake may disrupt vitamin D metabolism, potentially impairing its effectiveness and compromising maternal and fetal bone health (Rude et al., 2009b). Additionally, magnesium interacts with vitamin B6, a nutrient crucial for amino acid metabolism, neurotransmitter synthesis, and fetal brain development. Disruptions in vitamin B6 metabolism due to magnesium supplementation may have implications for maternal mood, cognitive function, and fetal neurodevelopment (Bender, 1994).

Furthermore, the interaction between magnesium and nutrients extends beyond absorption and utilization to include physiological processes within the body. For example, magnesium and potassium work synergistically to regulate muscle function and nerve transmission. However, an imbalance in magnesium levels can disrupt potassium homeostasis, leading to muscle weakness, cramps, and irregular heart rhythms (Jahnen‐Dechent & Ketteler, 2012). This highlights the importance of maintaining an appropriate balance of electrolytes, including magnesium and potassium, to support maternal cardiovascular health and prevent complications during pregnancy. Additionally, magnesium supplementation may impact the bioavailability of certain nutrients, altering their physiological effects within the body. For instance, magnesium binds to phytic acid, a compound found in grains and legumes that can inhibit mineral absorption. While magnesium supplementation may enhance the absorption of minerals bound to phytic acid, such as zinc and manganese, it may also decrease the bioavailability of other nutrients, such as calcium and iron (Lönnerdal, 2000). This underscores the complexity of nutrient interactions and the need for careful consideration when recommending magnesium supplementation during pregnancy. However, magnesium plays a critical role in maternal and fetal health during pregnancy; its interactions with other nutrients must be carefully considered to optimize outcomes. Nutrient absorption interference, utilization disruptions, and physiological imbalances resulting from magnesium supplementation can have significant implications for maternal and fetal well‐being.

## 11 CHALLENGES AND FUTURE DIRECTIONS

### 11.1 Challenges

- *Nutrient gaps*: Many pregnant women, especially in low‐income populations, still face challenges in accessing and affording nutrient‐rich foods and supplements. Addressing these disparities is crucial to ensure equitable maternal–fetal health outcomes (Makrides & Crosby, 2014).
- *Education and awareness*: A lack of awareness about the importance of micronutrients and a balanced diet during pregnancy remains a challenge. Comprehensive education campaigns and improved healthcare provider training are needed to enhance awareness (Doyle et al., 2009).
- *Compliance with prenatal supplements*: Compliance with prenatal supplements can be challenging due to the side effects, taste, and size of the supplements. Developing more palatable and user‐friendly supplements can improve adherence (Doyle et al., 2009).
- *Micronutrient interactions*: Understanding the complex interactions between various micronutrients during pregnancy is an ongoing challenge. Research should focus on elucidating these interactions to develop targeted supplementation strategies (Duley et al., 2010).
- *Optimal dosages*: Determining the optimal dosage of micronutrient supplements for specific populations and stages of pregnancy requires further investigation. One‐size‐fits‐all approaches may not be effective for all individuals (Singh, Fedacko, et al., 2022).
- *Monitoring and assessment*: Developing affordable and accessible methods for monitoring micronutrient status, particularly in resource‐limited settings, is critical for the early detection of deficiencies (Singh, Fedacko, et al., 2022).

### 11.2 Future directions

- *Personalized nutrition*: Advancements in genetics and biomarker research may pave the way for personalized nutrition recommendations during pregnancy. Tailoring dietary and supplementation advice to an individual's unique needs will become more feasible (Kovacs, 2016).
- *Micronutrient fortification*: Expanding fortification programs to include a broader range of essential micronutrients in staple foods can help address deficiencies at the population level (Tiwari et al., 2020).
- *Innovative supplements*: Continued research into innovative supplement delivery systems, such as micronutrient‐rich foods, gummies, or easily dissolvable tablets, can improve supplement compliance (Doyle et al., 2009).
- *Digital health solutions*: Leveraging technology for dietary tracking and reminders can enhance pregnant women's adherence to dietary and supplement recommendations (Makrides & Crosby, 2014).
- *Global collaboration*: Collaborative efforts among governments, nongovernmental organizations (NGOs), healthcare providers, and the private sector are crucial for ensuring access to affordable and high‐quality prenatal supplements and fortified foods (Doyle et al., 2009).
- *Maternal–fetal microbiome*: Exploring the role of the microbiome in nutrient absorption and utilization during pregnancy offers exciting avenues for research and interventions (Duley et al., 2010).

## 12 SUMMARY

Maternal–fetal health is a delicate interplay of physiological, nutritional, and environmental factors, with micronutrients playing a pivotal role in this intricate balance. From conception to childbirth, the journey of pregnancy is marked by profound changes and increased nutrient demands. Ensuring optimal maternal–fetal outcomes requires a comprehensive understanding of the significance of micronutrients and their interactions. Throughout this exploration, we have delved into the multifaceted world of micronutrients, emphasizing the importance of balanced nutrition during pregnancy. From the earliest stages of fetal development, folate and vitamin B12 contribute to the formation of a healthy neural tube, guarding against devastating birth defects. The dynamic duo of calcium and magnesium promotes skeletal strength and muscle function, while iron and vitamin C safeguard against anemia, ensuring an adequate oxygen supply to both mother and fetus. The synergy between micronutrients reveals itself in intricate relationships: vitamin D aids in calcium absorption, while magnesium enhances zinc uptake. Such interactions orchestrate countless physiological processes essential for fetal growth and maternal health. However, challenges persist. Disparities in nutrient access and awareness continue to affect maternal–fetal health outcomes, necessitating educational campaigns and targeted interventions. The future holds promise, with advancements in personalized nutrition, innovative supplementation strategies, and digital health solutions on the horizon.

In conclusion, nurturing maternal–fetal health through micronutrients is not merely a matter of dietary choice; it is a cornerstone of responsible prenatal care. From addressing nutrient deficiencies to embracing the potential of personalized nutrition, the journey to healthier pregnancies is an ongoing endeavor. With research, education, and global collaboration, we can pave the way for brighter, healthier beginnings for both mothers and their precious newborns. Maternal–fetal health is a shared responsibility, and the power of micronutrients is a beacon guiding the way toward a healthier, nourished future.

## AUTHOR CONTRIBUTIONS

**Vani Shukla:** Conceptualization (equal). **Sidrah Parvez:** Writing – original draft (equal). **Ghizal Fatima:** Writing – original draft (equal). **Shikha Singh:** Methodology (equal). **Aminat Magomedova:** Methodology (equal); resources (equal). **Gaber El‐Saber Batiha:** Resources (equal); writing – original draft (equal). **Athanasios Alexiou:** Methodology (equal); resources (equal). **Marios Papadakis:** Project administration (equal); supervision (equal); validation (equal). **Nermeen N. Welson:** Writing – review and editing (equal). **Najah Hadi:** Resources (equal); writing – review and editing (equal).

## FUNDING INFORMATION

Open Access funding was enabled and organized by Projekt DEAL. This work was supported by the University of Witten‐Herdecke, Germany.

## CONFLICT OF INTEREST STATEMENT

All the authors declare no conflict of interest.

## ETHICAL APPROVAL

This study does not involve any human or animal testing.

## Data Availability

All collected data are included in this review.
